# Power Doppler ultrasound and contrast-enhanced ultrasound demonstrate non-invasive tumour vascular response to anti-vascular therapy in canine cancer patients

**DOI:** 10.1038/s41598-019-45682-2

**Published:** 2019-06-25

**Authors:** Eline Abma, Emmelie Stock, Ward De Spiegelaere, Leen Van Brantegem, Katrien Vanderperren, Yicheng Ni, Matthijs Vynck, Sylvie Daminet, Kaat De Clercq, Hilde de Rooster

**Affiliations:** 1 Small Animal Department, Faculty of Veterinary Medicine, Ghent University, Salisburylaan 133, B-9820 Merelbeke, Belgium; 2 Cancer Research Institute Ghent (CRIG), Medical Research Building, University Hospital Ghent, De Pintelaan 185, B-9000 Ghent, Belgium; 3 Department of Medical Imaging and Orthopedics of Small Animals, Faculty of Veterinary Medicine, University of Ghent, Salisburylaan 133, B-9820 Merelbeke, Belgium; 4 Department of Morphology, Faculty of Veterinary Medicine, Ghent University, Salisburylaan 133, B-9820 Merelbeke, Belgium; 5 Department of Pathology, Bacteriology and Poultry Diseases, Faculty of Veterinary Medicine, Ghent University, Salisburylaan 133, B-9820 Merelbeke, Belgium; 6 Theragnostic Lab, Department of Imaging and Pathology, KU Leuven, Herestraat 49, B-3000 Leuven, Belgium; 7 Department of Data Analysis and Mathematical Modeling, Faculty of Bioscience Engineering, Ghent University, Coupure links 653, B-9000 Ghent, Belgium; 8 Laboratory of Pharmaceutical Technology, Ghent University, Ottergemsesteenweg 460, B-9000 Ghent, Belgium

**Keywords:** Cancer imaging, Diagnostics

## Abstract

Combretastatin A4-phosphate (CA4P) is an anti-vascular agent which selectively shuts down blood supply in tumours, resulting in extensive tumour necrosis. The aim of this study was to assess *in vivo*, non-invasive ultrasound techniques for the early evaluation of tumour perfusion following CA4P treatment of spontaneous tumours. Eight dogs that bore spontaneous tumours were enrolled and were subsequently treated with a single dose of intravenous CA4P. Perfusion of tumours was evaluated by power Doppler ultrasound (PDUS) pre-treatment (0 h), during the injection (10 min, 20 min, 30 min) and after CA4P infusion (24 and 72 h). Vascularity index (VI) of the tumour tissue was quantitatively analysed and accuracy was verified by correlation analysis with the results of immunohistochemical evaluation of microvessel density (MVD). Central and peripheral perfusion was evaluated by contrast-enhanced ultrasound (CEUS) pre-treatment and at 72 h post-treatment. Post-treatment, PDUS demonstrated a significant decrease in VI within 10 min of CA4P infusion. CEUS parameters demonstrated a significant decrease in blood velocity and volume in the central aspect of the tumour. Histology revealed a 4.4-fold reduction (p < 0.001, 95% CI [2.2,9.4]) in MVD and a 4.1-fold increase (p = 0.003, 95% CI [1.4,11.8]) in necrotic tumour tissue. A strong correlation between PDUS results and immunohistochemical results was found (Pearson R^2^ = 0.957, p < 0.001). Furthermore, the findings of PDUS were supported by the objective results of the CEUS analyses. These data suggest a role for ultrasound in real-time, non-invasive monitoring of tumour vascular response as an early indicator of CA4P treatment efficacy.

## Introduction

Tumour vasculature is crucial for survival, growth, and spread of tumours and is a promising pharmacological target in anti-cancer strategies^1,2^. Vascular targeting exploits the morphological differences between tumour and normal vasculature^3–9^. Whereas antiangiogenic agents (AAA’s) prevent formation of new blood vessels, vascular disrupting agents (VDA’s) destroy the tumour neovasculature^10,11^.

Combretastatin A4-phosphate (CA4P) is the best known VDA. It depolymerizes the microtubules of immature vessel endothelial cells, inducing vessel occlusion and resulting in shutdown of tumour perfusion within an hour of administration^6,12,13^. This drug has been subjected to a range of combined-therapy trials, including surgery, anti-angiogenic therapy, chemotherapy, and radiotherapy^14–21^. Considering the success of anti-vascular therapies and their future prospects, efficient screening methods of treatment response are highly sought-after.

The gold standard to assess the therapeutic effect of anti-vascular therapy on tumour vascularisation is immunohistochemical determination of microvessel density (MVD) (maximal number of blood vessels per unit area of section) in pre- and post-treatment surgical biopsies^22^. Given the invasive nature of sequential biopsies and the repeated need for anaesthesia, immunohistochemical MVD assessment has limited utility in a clinical setting. Thus, there is a clinical interest in non-invasive yet accurate imaging techniques that allow serial assessment of tumour perfusion *in situ*, enabling visualization of therapy response.

Current non-invasive imaging modalities to effectively evaluate the vascular response to CA4P and other VDA’s in rodents, rabbits, and men include dynamic contrast-enhanced magnetic resonance imaging (DCE-MRI)^23–28^, diffusion-weighted imaging^25,29^, perfusion computed tomography^30^, positron emission tomography (PET)^31^, bioluminescence imaging (BLI)^26,28,32,33^, photoacoustic imaging (PAI)^34,35^, and dynamic contrast-enhanced (DyCE) fluorescent imaging^36^. Recently, contrast-enhanced ultrasound (CEUS) assessment in tumour-bearing mice was added to this list^26,37–39^. Power Doppler ultrasound (PDUS) has been used to assess vascularity in a range of animal and human tumours^22,40–43^ and for detection of blood flow changes following anti-vascular treatment^32,44–46^. It was recently found to be a reliable technique for the *in vivo* assessment of tumour angiogenesis^41^, but it has not yet been evaluated if this modality can be used as a reliable, non-invasive alternative to biopsies to asses CA4P treatment response. Considering that CA4P has been shown to reduce tumour blood flow within the first hour of therapy, well before vessels can regress, PDUS might provide a more veracious assessment of early response than immunohistochemistry.

In this study, we performed PDUS to assess the vascularity index (VI) before, during, and after a single dose of intravenous CA4P in a variety of spontaneous superficial solid tumours in dogs. Additionally, we used CEUS to quantitatively evaluate the intratumoural perfusion, both in the central and peripheral regions of the tumours. To verify the accuracy of PDUS, a correlation analysis was performed on the PDUS results and the results of conventional MVD determined by immunohistochemistry, at 72 h post-treatment.

The aim of this work was to conduct a proof-of-principle study to assess the feasibility of using PDUS and CEUS as methods for assessing early tumour vascular inhibition by CA4P in clinical studies involving spontaneous, superficial solid tumours.

## Results

### Patient characteristics

Eight dogs entered the study. Baseline characteristics of participating patients are summarized in Table 1 and are further described elsewhere^45^. The study cohort consisted of four males and four females, belonging to six different breeds, and included five mesenchymal tumours, two epithelial tumours, and one round-cell tumour.

**Table 1:** Patient characteristics and tumour type of the dogs treated with combretastatin A4-phosphate.

Dog n°	Characteristics (breed, sex, age, weight)	Tumour type, localization, size
1	Mixed breed, F, 7.5 y, 13 kg	Adenocarcinoma, mammary gland, 30.2 cm^3^
2	American Staffordshire, Mn, 14.1 y, 36 kg	Soft tissue sarcoma, right carpus, 252.7 cm^3^
3	Golden Retriever, Mn, 10.7 y, 31 kg	Chondrosarcoma, left elbow, 339.6 cm^3^
4	Münsterlander, Fn, 4.8 y, 21 kg	Soft tissue sarcoma, perineal, 897.2 cm^3^
5	American Staffordshire, Mn, 10.0 y, 38 kg	Mastocytoma, left carpus, 112.5 cm^3^
6	Whippet, Fn, 7.8 y, 13 kg	Soft tissue sarcoma, right carpus, 0.6 cm^3^
7	Mixed breed, F, 8.4 y, 12 kg	Adenocarcinoma, mammary gland, 0.7 cm^3^
8	Bernese mountain dog, Mn, 7.5 y, 38 kg	Histiocytic sarcoma, facial, 25.0 cm^3^

Abbreviations: F, Female; Fn, Female neutered; M, Male; Mn, Male neutered; y: age in years.

### Tumour vascularity as assessed by power Doppler ultrasound

**Figure 1:**
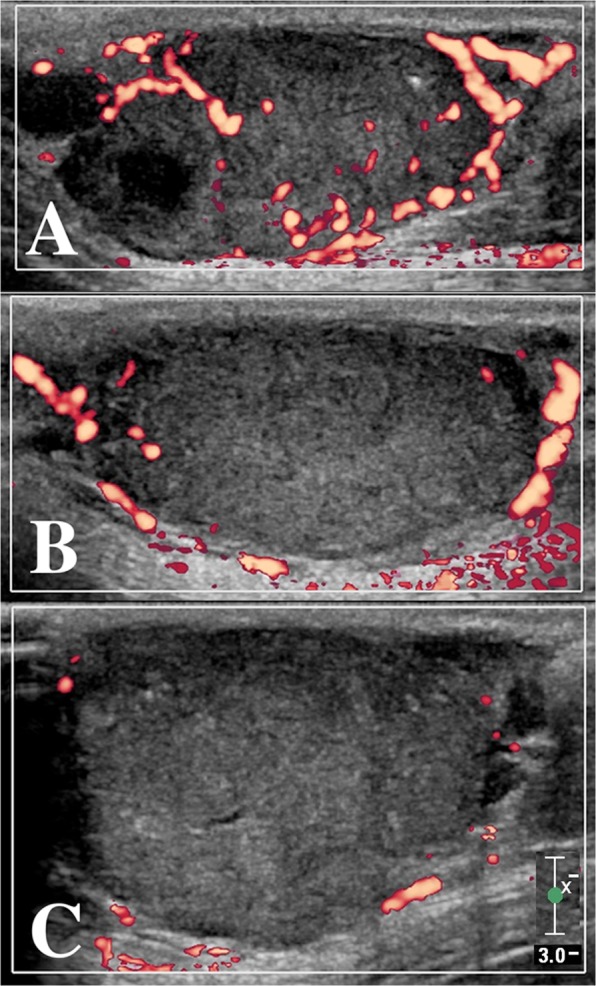
Tumour vascularization as measured by power Doppler ultrasound of a mammary gland adenocarcinoma (dog 1). (**A**) before intravenous CA4P treatment, (**B**) 24 h after treatment, (**C**) 72 h after treatment at matched locations. A decrease in central tumour vascularization is clearly noticeable (Scale bar = 3 cm).

Pre-treatment (pre-CA4P), there appeared to be a marked variability in the tumour perfusion as evaluated by PDUS (Table 2). The sarcomas were less well vascularized pre-treatment than the carcinomas and the mastocytoma. After 10 min of CA4P infusion, an overall significant decrease in VI was measured (p = 0.039) (Fig. 1). Although not every VI continued to decrease to the same extent, and three VI’s even demonstrated a slight increase between 24 and 72 h post-infusion (PI), overall the decrease in VI remained significant until 72 h PI.

**Table 2:** Vascularity index and CEUS parameters *in vivo*, and the relative amount of necrotic tumoural tissue and microvessel density *ex vivo*, pre-treatment and 72 hours after intravenous CA4P administration (75 mg m^−2^) in dogs with cancer

Dog n°, tumour type	PDUS						CEUS										Histology			
	Vascularity Index (Vessels tumour area^−1^)						CEUS Parameters (Central tumour *in vivo*)										Necrotic tumoural tissue (Necrosis tumour area^−1^)		Microvessel density (Vessels tumour area^−1^)	
	(*In vivo*)						PE (au)		WiAUC (au)		TTP (s)		RT (s)		mTT (s)		*(Ex vivo)*		*(Ex vivo)*	
	base	10 min	20 min	30 min	24 h	72 h	Base	72 h	Base	72 h	Base	72 h	Base	72 h	Base	72 h	base	72 h	base	72 h
1, C	0.48	0.37	0.23	0.14	0.10	0.04	3536	229	11553	2618	15.22	50.29	8.84	15.98	52.92	110.78	0.015	0.110	0.54	0.03
2, S	0.17	0.11	0.09	0.09	0.04	0.07	3247	1021	15600	8340	18.38	31.82	7.87	14.74	30.09	64.05	0.001	0.215	0.21	0.06
3, S	0.15	0.12	0.06	0.03	0.03	0.03	14	1	91	3	16.89	18.00	7.66	11.22	6.60	51.96	0.012	0.853	0.09	0.02
4, S	0.04	0.04	0.03	0.01	0.02	0.01	1357	610	4933	2760	14.27	16.53	5.90	7.34	32.37	50.06	0.013	0.023	0.03	0.02
5, M	0.26	0.23	0.16	0.20	0.20	0.24	2535	363	19967	1657	20.08	31.15	3.58	17.51	31.79	60.17	0.009	0.014	0.27	0.24
6, S	0.23	0.20	0.21	0.19	0.16	0.20	2170	2757	12940	25567	24.13	23.73	9.46	10.06	34.99	36.12	0.180	0.110	0.22	0.19
7, C	0.30	0.28	0.23	0.18	0.09	0.04	5407	3953	24967	20033	22.05	20.09	11.95	8.17	49.37	48.55	0.027	0.613	0.29	0.02
8, S	0.19	0.17	0.13	0.12	0.10	0.05	396	68	1374	185	19.40	38.21	3.52	9.18	52.50	61.41	0.158	0.404	0.20	0.06

Abbreviations: CEUS, Contrast-enhanced ultrasound; CA4P, Combretastatin A4-phosphate; PDUS, Power Doppler ultrasound; PE, Peak enhancement; AU, Arbitrary unit, WiAUC, Wash-in area-under-curve; TTP, Time to peak; RT, Rise time; mTT, Mean transit time; C, Carcinoma; S, Sarcoma; M, Mastocytoma.

### Quantitative analysis of contrast-enhanced ultrasound

**Figure 2:**
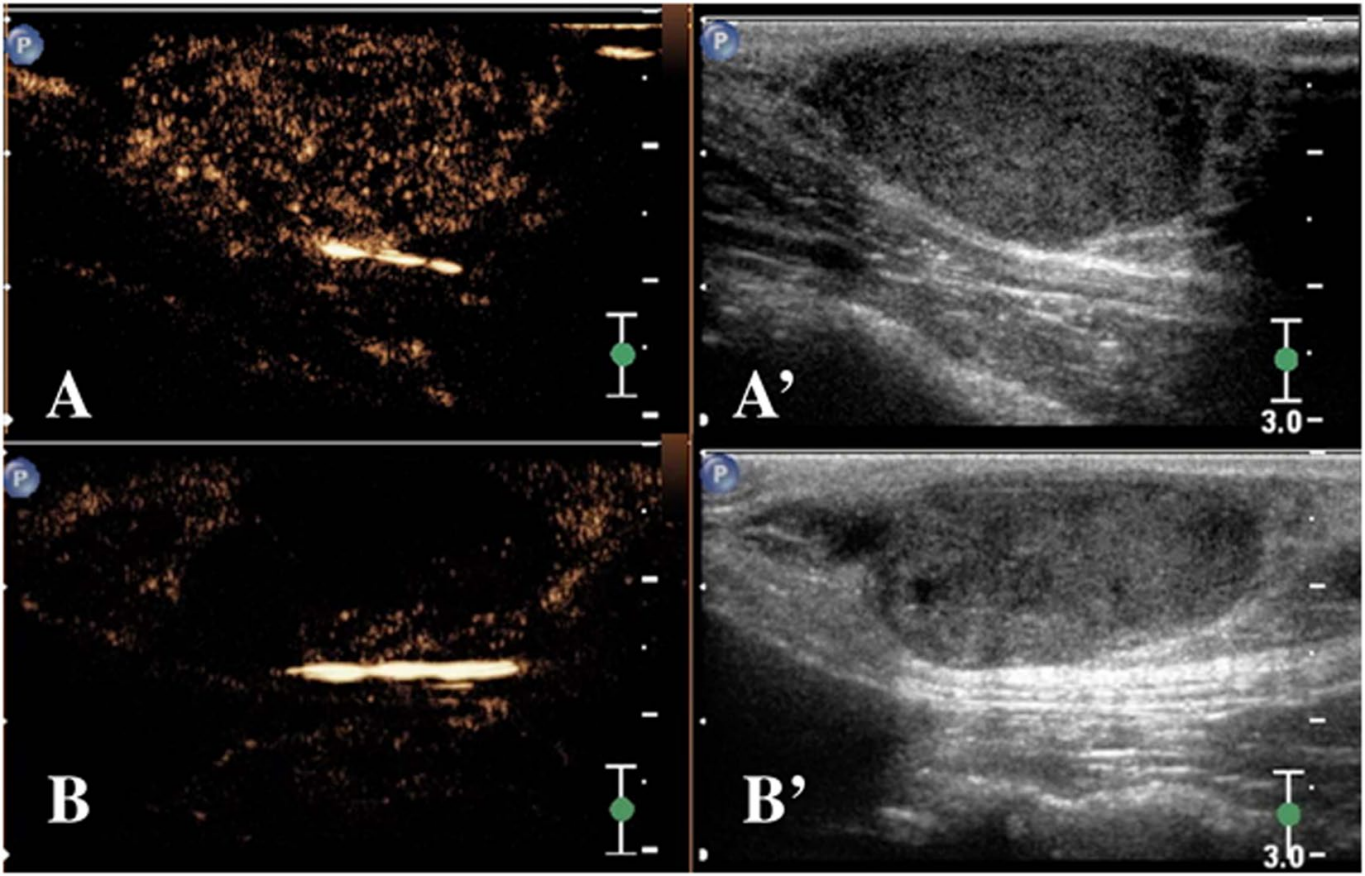
Tumour evaluation after CA4P treatment using contrast-enhanced ultrasound (CEUS) of a mammary gland adenocarcinoma at a matched location (dog 1). The left side of the image corresponds to the CEUS image of the tumour, the right side to the B-mode ultrasound. (**A**) before intravenous CA4P treatment, (**B**) 24 h after treatment. There is a large diffuse uptake of microbubble contrast throughout the entire tumour before treatment. In contrast, 24 h after treatment, uptake of microbubble contrast has greatly diminished in the central tumour, indicating a decrease in central tumour vascularization (Scale bar = 3 cm).

**Figure 3:**
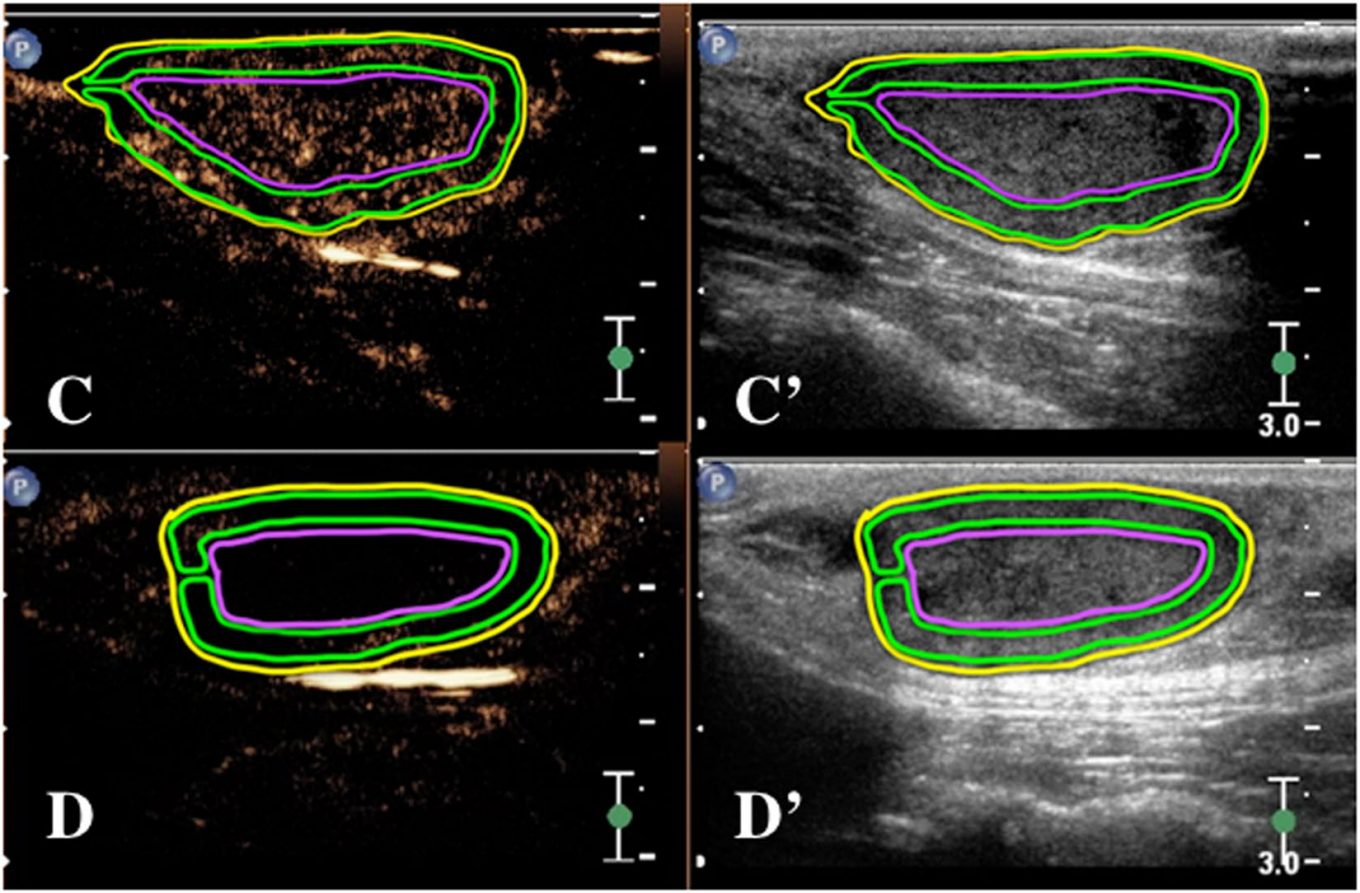
Regions of interest were manually drawn on the contrast-enhanced ultrasound (CEUS) image of Fig. 2: Yellow encompasses the entire tumour, green outlines the peripheral one-third radius, and magenta contains the centre two-thirds radius of the tumour. (**C**) Before intravenous CA4P treatment, (**D**) 24 h after treatment (Scale bar = 3 cm).

**Figure 4:**
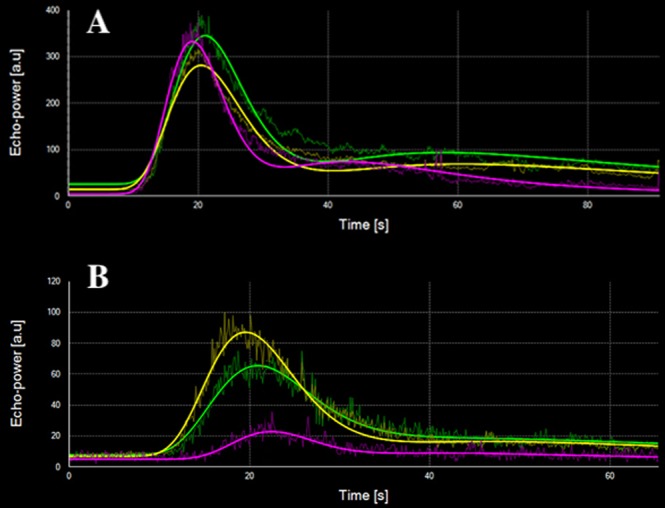
Tumour evaluation after CA4P treatment of a mastocytoma using contrast-enhanced ultrasound (CEUS) of 3 regions of interest (ROI) (dog 5). Time-intensity curves of ROI_*whole*_ (yellow), ROI_*periphery*_ (green), and ROI_*centre*_ (magenta). (**A**) before treatment, (**B**) 72 h after treatment. An obvious decrease in tumour vascularization is noted by CEUS in all three ROI’s, but is most prominent in tumour centre.

Tumour perfusion parameters assessed by CEUS also revealed the sarcomas to be less well vascularized pre-treatment than the carcinomas and the mastocytoma (Table 2). A difference in blood flow parameters of the tumour centre was evident in all tumour types after CA4P administration (Figs 2–4). Overall, a significant decrease in tumour blood volume [characterized by the peak enhancement (PE) (p = 0.001) and wash-in area-under-curve (WiAUC) (p = 0.001)] was observed after CA4P administration. Furthermore, a significantly slower blood flow velocity [characterized by the time to peak (TTP) (p = 0.003) and rise time (RT) (p = 0.006)] were present. Additionally, a significant increase (p = 0.019) of the residence time of blood in the tumour [characterized by the mean transit time (mTT)] was noted. Interestingly, blood flow parameters at the tumour periphery did not significantly alter after treatment in any of the dogs.

### Histopathological assessment of tumour necrosis and microvessel density

Pre-treatment and post-CA4P results for all patients are listed in Table 2.

**Figure 5:**
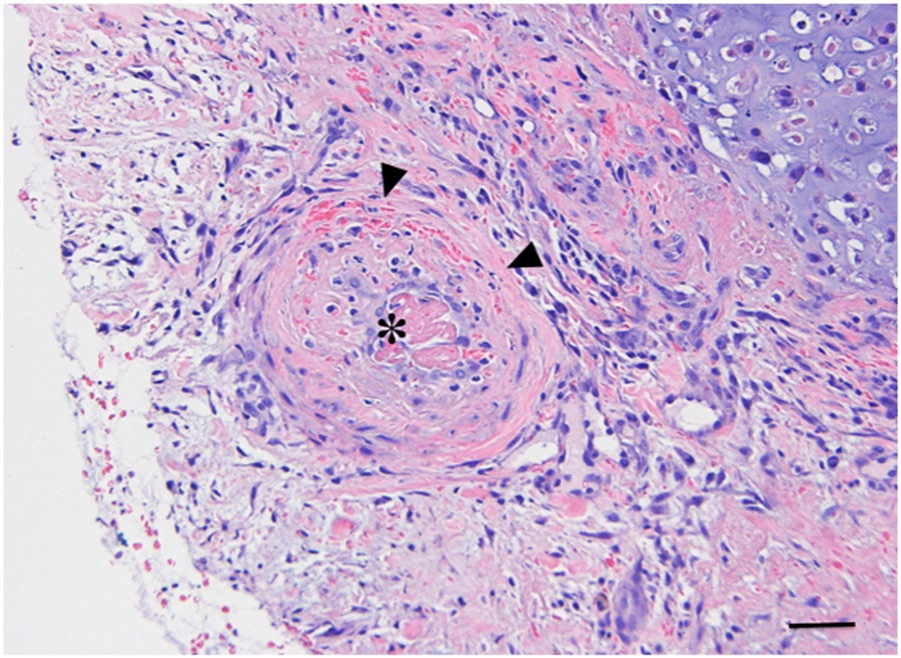
Histopathological section of the central part of a chondrosarcoma (dog 3), 72 h after intravenous CA4P administration. The vessel has been obstructed by a thrombus (asterisk) and necrosis of the endothelial cell lining (arrowheads) is visible (Haemotoxylin and Eosin. Scale bar = 50 µm).

Areas of necrosis were characterized by loss of tissue architecture, eosinophilic cellular debris, pyknosis of nuclei and/or nuclear fragmentation. The estimated overall ratio for necrosis was 0.08 (95% CI [0.02 to 0.19]) before treatment and 0.33 (95% CI [0.01 to 0.82]) 72 h after treatment, corresponding to an overall 4.1-fold increase in necrosis of the tumour after a single dose of CA4P (p = 0.003, 95% CI [1.39 to 11.81]). Several tumours demonstrated a heterogeneous response within one biopsy sample. Areas of necrotic cells were interspersed with areas of mitotic cells. In some tumours, large areas of the tumour had undergone complete cellular degeneration and only a few viable cells remained. These viable cells were arranged concentrically around the remaining patent blood vessels. In contrast, vessels that were blocked, resulting in complete vascular occlusion, were surrounded by necrotic tumour cells. The endothelial cells of these obstructed vessels were fragmented and in several of the blocked tumour vessels, thrombi were observed. (Fig. 5).

**Figure 6:**
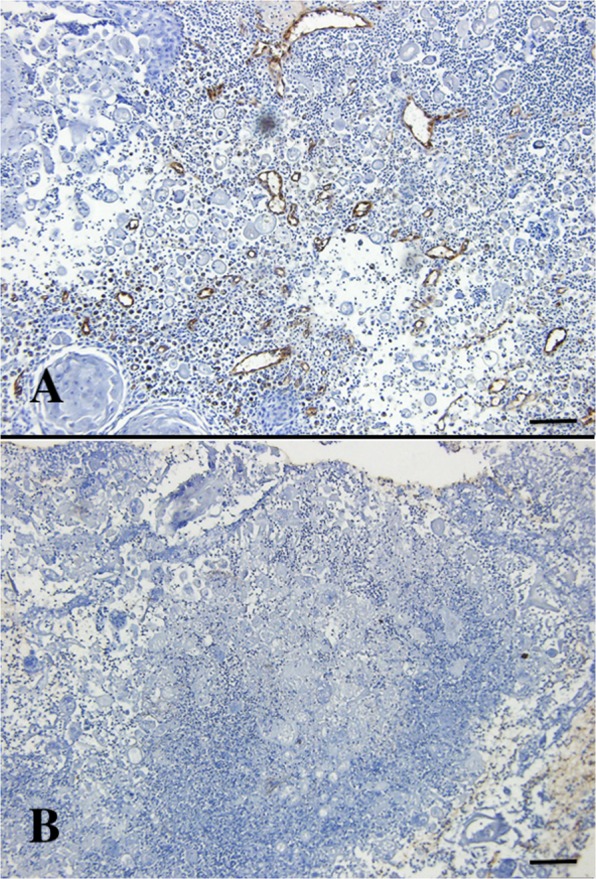
Microvessel density counting in a mammary gland adenocarcinoma (dog 7), (**A**) Two weeks before CA4P treatment, (**B**) 72 h after CA4P treatment. A decrease in microvessel density is clearly noticeable (Anti-von Willebrand Factor. Scale bar = 100 µm).

Concerning the MVD, the sarcomas demonstrated a lower MVD pre-treatment than the carcinomas and the mastocytoma, supporting the CEUS and PDUS findings. The number of tumour microvessels, identified by the von-Willebrand factor (vWF) staining, was observed to have decreased significantly 72 h after treatment (Fig. 6). The estimated ratio of MVD was 0.22 (95% CI [0.09 to 0.36]) pre-treatment versus 0.05 (95% CI [0.03 to 0.08]) post-treatment, corresponding to a 4.4-fold reduction (p < 0.001, 95% CI [2.23 to 9.39]) in tumour microvessels 72 h after a single dose of CA4P.

### Correlation of ultrasound parameters with (immuno)histopathological findings

**Figure 7:**
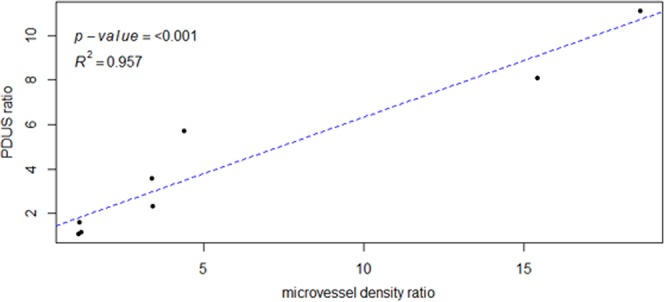
Scatter plot revealing the correlation between the power Doppler ultrasound (PDUS) and the microvessel density (R^2^ = 0.957; p < 0.001). The X- and Y-axes are expressed as the ratios between pre- and post CA4P measurements.

For both the treated and pre-treated dogs, correlations between PDUS and MVD and between CEUS and MVD are illustrated in Fig. 7. Power Doppler ultrasound was highly correlated with the MVD determinations on the anti vWF staining (Pearson R^2^ = 0.957, p < 0.001). There was no significant correlation between any of the CEUS parameters and the MVD: PE (R^2^ = 0.397, p = 0.33), WiAUC (R^2^ = −0.166, p = 0.695), TTP (R^2^ = −0.217, p = 0.605), RT (R^2^ = 0.395, p = 0.333), and mTT (R^2^ = 0.078, p = 0.854).

## Discussion

In this study, PDUS was compared with histopathology and assessed as a technique for the *in vivo* assessment of spontaneous tumour vascular response to CA4P treatment. Additionally, the reduction in tumour blood velocity was assessed by means of CEUS. Power Doppler ultrasound (US), CEUS and histopathology each revealed vascular shutdown with considerable necrosis within a 72 h period post-treatment.

Ideally, clinical monitoring tools should be non-invasive, allowing for serial assessments of the same tumour mass without risk for the patient. Currently, the gold standard to assess the therapeutic effect of anti-vascular therapy on tumour vascularisation is microscopic determination of MVD in pre- and post-treatment histological tissue sections^22^. To improve blood vessel detection on histological slides, immunohistochemical staining of endothelial cell markers is performed. However, in addition to tissue biopsy being an invasive monitoring technique, early treatment response of anti-vascular agents such as CA4P can be misjudged in post-treatment biopsies; as several VDA’s induce changes in tumour blood perfusion well before vessel regression becomes apparent on histopathological tissue sections^22^.

The need for non-invasive techniques allowing early evaluation of clinical patients treated with fast-acting anti-vascular treatments is evident. To date, non-invasive techniques such as DCE-MRI, PET, and near-infrared spectroscopy have been explored to demonstrate changes in tumour perfusion and viability in both rodent tumour models and human patients^13,23,27,47^. However, these techniques are not always readily available and they are expensive, and some require ionizing radiation (PET) and/or heavy sedation or even general anaesthesia of the patient (DCE-MRI, PET). In contrast, US is assumed to be a promising angiogenesis assessment modality, as it is a widely available, non-radioactive, real-time and cost-effective alternative for above-mentioned techniques^39^. Additionally, CEUS is also suitable for use in patients with renal and hepatic failure because of the pulmonary elimination of the contrast agent^48^.

Vascular imaging such as US allows quantification of the number and spacing of blood vessels and to assess blood flow in a non-invasive and functionally relevant way. Therefore, US has the potential to assess the immediate efficacy of anti-vascular therapy^49^. Power Doppler US is a technique that displays the strength of the amplitude in colour, rather than speed or direction information, by using the amplitude of a Doppler shift to detect moving matter^50^. Noise is assigned a uniform colour that remains constant while flow is shown as a contrasting colour. As a result, PDUS has three to five times the sensitivity of conventional colour Doppler for the detection of flow^50,51^. Although the sensitivity of the technique certainly varies with both vessel calibre and flow rate, previous studies have demonstrated Doppler detection of flow through tumour vessels as small as 10–20 μm^52,53^.

The results of the PDUS clearly confirmed that CA4P treatment of spontaneous solid tumours in dogs resulted in an acute tumour vascular response within 10 min after start of the infusion. The statistically significant correlation between intratumoural VI and the gold standard MVD at 72 h supports the hypothesis that PDUS offers an objective and reliable assessment of the tumour vascularity at the time of US scanning in an *in vivo* and *in situ* manner^22,41,43,44^. Standardized power Doppler settings and image-processing protocols enable a precise and objective evaluation of the VI and make PDUS a convenient and immediately applicable way to determine the efficacy of anti-vascular agents in a therapeutic setting.

The susceptibility of PDUS to flash artefacts, because of its high motion sensitivity, is one of its major limitations. Patient cooperation is necessary to decrease artefacts resulting from excessive movement (e.g. panting), as prominent soft-tissue motion may preclude the use of PDUS. For human patients, this is not likely to form a problem; veterinary patients may need sedation with drugs known not to interfere with vascularisation (i.e. Butorphanol, MSD, Brussel, Belgium)^54^ if highly excited or fearful.

The CEUS contrast agent SonoVue^TM^ consists of sulfur hexafluoride-filled microbubbels (MB’s) in a phospholipid shell^48^. The mean diameter of the sulfur hexafluoride-filled MB’s is 2.5 µm, resulting in a strictly intravascular trajectory of the bubbles without any interstitial component^55^. At low acoustic power, alternating expansion and contraction of the MB’s result in a vibration, generating harmonic echoes. The harmonic signal produced by the MB’s is greater than that of the surrounding tissue, making CEUS a very sensitive imaging technique, well suited for the evaluation of blood flow in functional vessels^56^.

After CA4P administration, all tumours demonstrated significant changes in the CEUS perfusion characteristics. Pre-treatment, CEUS demonstrated a rapid inflow of the MB’s into the entire tumour, a distinctive feature of malignant tumours^55,57^, followed by a slow clearance. Post-treatment, the time constant for contrast to reach the tumour (TTP) was increased, likely indicating a less aggressive behaviour of the tumour after CA4P treatment. Together with a significant drop in tumour blood volume (PE and WiAUC) and increase in tumour transit time (RT and mTT), it is possible that CA4P treatment induced a slower tumour progression, despite a continued growth of the tumour if no adjuvant therapy is administered^58^. Of particular interest was that the software for analysis of the CEUS data allowed for an easy and objective distinction between the measurements of tumour centre versus periphery, which is important in the determination of drug efficacy and prognosis. Results post-CA4P-treatment clearly demonstrated a high susceptibility of the tumour centre to necrosis formation while the perfusion of the tumour periphery was consistently poorly affected, which is in accordance with previous research^3,6,47,59^.

Although the CEUS parameters demonstrated significant reduction in perfusion in the tumour centre, the correlation between the CEUS parameters and the gold standard MVD failed to reach statistical significance. In the study carried out by Zhang and colleagues^39^, the CEUS parameter maximum intensity (IMAX) was found to be closely correlated with MVD. This parameter represents the average US contrast agent concentration of the analysis region of interest (ROI) (tumour tissue) relative to that of the reference ROI (healthy tissue) which is assumed to be 100%. Calculating the IMAX necessitates obtaining images including both tumour tissue and healthy tissue in the same image. This is perfectly feasible in rodent tumour models, with tumours reaching only several mm’s in diameter. However, in pet animal or human patients with spontaneous tumours, which can easily reach several cm’s in diameter, it is not possible to obtain images containing both tumoural and healthy tissue. Additionally, for some tumours it can be difficult to distinguish healthy tissue from tumour infiltrated tissue, based on US alone. Therefore, IMAX is not always a useful parameter in a clinical setting. Notwithstanding the fact that the CEUS parameters assessed in the current research were not significantly correlated to the MVD results, previous research demonstrated that CEUS provides an excellent non-invasive quantitative and qualitative alternative for tissue biopsies, as it has proven to be at least as sensitive a method for tumour angiogenesis monitoring as histopathology^39,60^. In this research, CEUS was not considered a reliable alternative, but rather an excellent adjunct to PDUS to assess tumour blood velocity between the different tumour areas before and after CA4P treatment. The necessity for intravenous administration and the availability of the MB’s and the dedicated software can be considered as drawbacks for the use of CEUS. However, the authors are of the opinion that the addition of CEUS to PDUS is relevant and beneficial, and the combination of these imaging modalities is an attractive and preferable alternative to sequential biopsies for the evaluation of CA4P treatment response.

There are some limitations to this clinical trial. Only a small number of patients with a variety of tumour types and stages were assessed. Additionally, only dogs with superficial tumours were enrolled in this proof-of-concept study because a superficial location made it practical and acceptable to obtain a 72 h post-treatment tumour biopsy. Lastly, due to ethical regulations, only tumoural tissue was biopsied and imaged by PDUS and CEUS, while healthy tissue was not evaluated. Therefore, it cannot be confirmed that the effect of CA4P was truly limited to tumoural tissue without healthy tissue being affected at all.

## Conclusion

This research confirms that measurement of the intratumoural VI by means of PDUS is correlated highly with the conventional MVD assay and is potentially useful for clinical monitoring of CA4P treatment. Assessment of the CEUS parameters support these findings and additionally prove primary response location to be the tumour centre. Additionally, our results emphasize that dogs with spontaneous cancer offer a novel and relevant model for studies of vascular disrupting therapy.

Considering the US results of this study, the clinical value of this non-invasive modality warrants further exploration and development of this technology during assessment of vascular targeting therapies.

## Methods

### Dogs

Dogs with histopathologically confirmed, superficial and well-defined solid tumours, suitable for US examination, were enrolled. Specifications on patient selection have been described elsewhere^45^.

Approval was obtained from the local research Ethical Committee (approval no. EC2015/143 and EC2016/66) of the Faculty of Veterinary Medicine of Ghent University, Belgium and from the Deontological Committee of the Federal Public Service of Health, Food Chain Safety and Environment, Belgium. The study protocol adhered to the guidelines and regulations of the *European Communities Council Directive (86/609/EEC)*/*principles of laboratory animal care (NIH publication No*. *85-23*, *revised 1985)*. Written informed consent was obtained from all patient owners before entry into the study. The use of CA4P in canine patients was approved (approval number 0002588) by the Belgian Federal Agency for Medicines and Health Products (FAMHP).

### Histologic tumour assessment

Pre-treatment tumour assessment consisted first of histopathological necrosis assessment and immunohistochemical MVD evaluation. A preliminary PDUS determined the imaging plane with maximum tumour perfusion and subsequently, after patient sedation, at least dual 6-mm punch biopsies were taken in this region. Further evaluation by medical imaging was then postponed for a minimum of two weeks, to preclude any influence (i.e. presence of air or suture material in the tumour) caused by the biopsy taking. 72 h following the CA4P-treatment and after all other assessments were completed, 6-mm punch biopsies were taken in the vicinity of the pre-treatment biopsies.

### Preparation and administration of sterile CA4P solutions

Specifications on the preparation and administration of CA4P have been described in our previous study^45^. Lyophilized CA4P (GMP quality) with a purity of >99% was purchased from Selleck Chemicals LLC (Houston, Texas, USA), and stored under light exclusion at a temperature of −20 °C. For the preparation of every personalized solution, the drug was accurately weighed (Mettler Toledo XS 204; readability 0.1 mg) and reconstituted in a sterile phosphate buffered saline (PBS) (Thermo Fisher Scientific, Erembodegem, Belgium) solution. Subsequently, the required volume of CA4P solution was pipetted and transferred to sterile amber vials. Finally, PBS was added until a final volume of 10 mL was reached. All CA4P solutions were prepared in a biological safety cabinet and were administered within 30 min of preparation as an intravenous infusion at a dose of 75 mg m^−2^ ^61^, over a time span of 30 min using a volumetric pump.

### Power Doppler ultrasound imaging

To obtain the PDUS images, a linear 12-5 MHz transducer was used on a dedicated ultrasound machine (Philips, iU22 xMATRIX, Philips Medical Systems, Bothell, WA, USA), operated at a Doppler gain of 76%, low persistence, pulse repetition frequency 500 Hz, and wall filter 47 Hz. The depth and position of the focus (single focus) were adapted based on the size of the tumour. The settings were standardized and repeated at each imaging session. The plane with maximum vascular signals was found by slowly moving the probe over the tumour, both in longitudinal and transverse orientation. The colour window was reduced in size to just cover the tumour to improve the colour sensitivity and frame rate. Stable vascular images and movies were captured and stored for quantitative analyses and the scan plane was documented to enable future scanning at the identical location. During the CA4P infusion, PDUS was performed at the identical tumour location at 10, 20 and 30 min. The PDUS examinations were repeated at 24 and 72 h PI at matched locations. At a later occasion, the captured images were retrieved and the boundary of the tumour was manually delineated on the computer with the use of the software Fiji^®^^62^. The density of the vascular signals within the demarcated tumour was then calculated and expressed as the VI (the number of vessels in demarcated tumour to total area of demarcated tumour) by a researcher blinded to patient identification and tumour treatment status.

### Contrast-enhanced ultrasound imaging

As described in our previous study, to perform the CEUS, a MB contrast agent (Sonovue^®^, Bracco, Milan, Italy), consisting of lipid-stabilized MB’s with a sulphur hexafluoride gas core, was injected IV as a bolus (0.04 mL kg^−1^) immediately followed by a 2.5 mL flush of sterile saline (Mini-Plasco NaCl 0.9%, Braun, Diegem, Belgium)^45^. Images were acquired using a linear 12-5 MHz probe. Machine settings were standardized: gain optimized to start with a nearly black baseline image (suppression background signal), dynamic range 50, mechanical index 0.09, and persistence off. The depth and position of the focus (single focus) were adapted based on the size of the tumour. During pre-treatment scanning, the scan plane was recorded for future assessment. All dogs received three injections of contrast media. Based on pre-existing knowledge, the second and third injection results were used since they resulted in more consistent enhancement than the first injection^63^. Between injections, the output power was adjusted to maximum with continued imaging of the tumour until background echogenicity was similar to pre-injection levels, indicating destruction of most residual contrast bubbles. In our experience, complete wash-out of the MBs was accomplished within 120 s, therefore the time frame for data collection was set at 0–120 s.

After CA4P infusion, CEUS was repeated at a matched tumour location at 72 h PI. All CEUS studies were digitally registered as movie clips (10 frames/sec) for two min after bolus injection. Clips were then exported to an offline computer, where they were randomized and anonymized. Subsequently, the clips were analysed using integrated dedicated specialized computer software (VueBox^®^, Bracco Suisse, Geneva, Switzerland) for objective quantitative analysis. Per study, three tumour ROI (whole tumour, tumour periphery, and tumour centre) were drawn manually on the CEUS image (Fig. 3). The first ROI encompassed the entire tumour. Second, the analysis ROI of the tumour periphery was drawn to outline the outer one-third radius of the tumour. Finally, the analysis ROI of the tumour centre was manually drawn inside the two-thirds radius of the tumour. Subsequently, mean pixel intensities and time-intensity curves were created for each ROI. The curves were then analysed for perfusion parameters representing blood volume and blood velocity.

### Histopathological analysis and immunohistochemistry

The tumour tissue samples were fixed in a 10% buffered formalin solution, embedded in paraffin, and sectioned at 4 μm. Per biopsy, four sections were cut at different depth within the biopsy to acquire technical replicate sections. Two sections with a 12 μm interstice were stained with Haematoxylin and Eosin (HE) for the histopathological evaluation of necrosis. For evaluation of the MVD, two sections with a 12 μm interstice were stained with the endothelial cell marker (vWF). Deparaffinized and hydrated sections underwent pre-treatment by 15 min incubation with Proteinase K S300402-2 (Agilent, Santa Clara, CA95051, USA) and subsequently by 5 min incubation with H_2_O_2_ solution to block the endogenous peroxidase activity. After rinsing, the sections were incubated with the primary antibody, i.e. polyclonal rabbit-anti human vWF antibody A0082 (Agilent, Santa Clara, CA95051, USA) was applied at 1:1600 dilution for 30 min at room temperature. Subsequently, HRP was linked to the primary antibodies using Envision Link Rabbit kit K401111-2 (Agilent, Santa Clara, CA95051, USA). Sections were then rinsed, incubated with 3,3′-diaminobenzidine solution, and counterstained with Haematoxylin.

**Figure 8:**
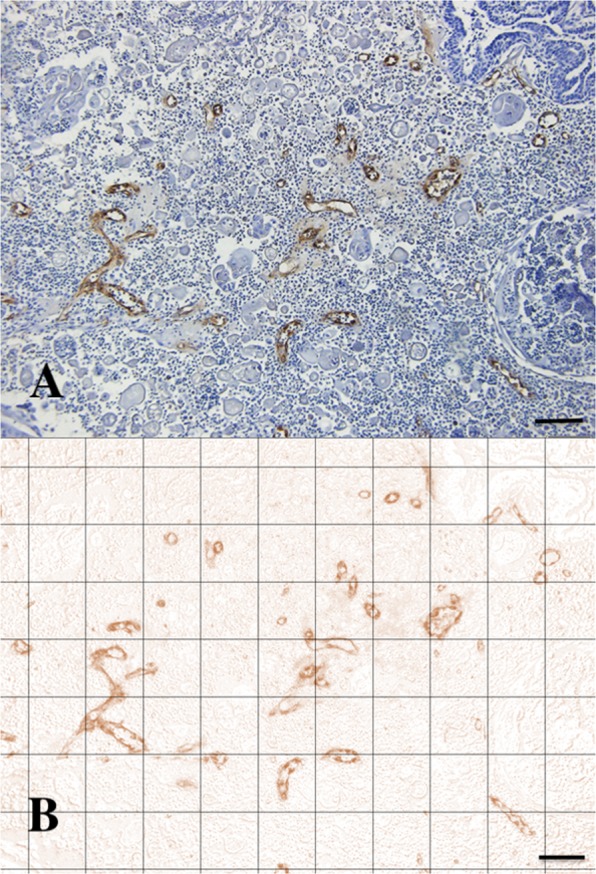
A representative slide of microvessel density counting in a mammary gland adenocarcinoma (dog 7) before treatment with CA4P, before (**A**) and after (**B**) application of an H-DAB filter (Anti-von Willebrand Factor. Scale bar = 100 µm).

Histological assessment of all biopsies was performed with the use of a Leica light microscope (Leica DMLB®, Leica Microsystems, Germany). Slides were first scanned at low power (×20) to identify tumoural tissue. Five fields within tumoural tissue per section were then selected randomly as counting frame at higher power (×100). Density measures were obtained by overlaying a grid on the counting frame and quantifying the crossing points that hit the area of interest, with the use of the Fiji^®^ software. In every HE-stained section, the relative amount of necrotic tumour tissue was expressed as the ratio of necrotic tumour tissue to the total tumour area. The averages of the five ratios were then calculated for each tumour biopsy. Microvessel counts were calculated in every vWF-stained section, based on the total number of vWF-positive structures within the tumour area and expressed as the MVD (ratio of tumour blood vessels mm^−2^ of tumour tissue). The averages of the five MVDs were then calculated for each tumour biopsy. Only uninterrupted round vWF-positive structures containing a lumen were counted as microvessels. Larger vessels with thick muscular walls and vessels with a lumen greater than approximately eight red blood cells were excluded, as suggested by Bosari and colleagues^64^. All measurements were performed blinded to patient identification and tumour treatment status. To facilitate recognition of vWF-positive structures, a H-DAB filter was applied within the FiJi^®^ software. Figure 8 shows a representative case of MVD counting.

### Statistical analysis

The statistical analyses were performed as described previously^45^. Except for the histological data, all data were analysed with nonparametric paired tests due to the non-normal distribution of the data using R (version 3.4.1). The Wilcoxon signed rank test and Friedman’s test (package stats, version 3.3.1) were used for comparing paired samples across two or more time points respectively. The Mack-Skilling’s test was used as replicated measures variant of the Friedman’s test accounting for multiple replicates per sample in the CEU data (package asbio, version 1.5). Post hoc analysis of Friedman’s tests was performed using Wilcoxon signed rank tests and all p-values were corrected using the Holm-Bonferroni method (package stats, version 3.3.1). A two-sided p-value of ≤0.05 was considered to indicate statistical significance.

Also the histological data, i.e. necrosis and blood vessel density data, were analysed as described in our previous study^45^, using generalized linear mixed models to account for variability of individual patients and biopsies using random effects. The package lme4 (version 1.1) was used in R (version 3.4.1). Given evidence of overdispersion, negative binomial generalized linear mixed models were fitted with the number of blood vessels or the necrosis surface as an outcome, treatment as a fixed effect, along with a random intercept and random slope to allow for differences in treatment effect between individual patients and control for correlation among replicated measurements. An offset was added to control the reference area of tumour tissue assessed in each counting frame.

The Pearson correlation coefficient was calculated and evaluated for statistical significance (package stats, version 3.3.1) to examine the correlation between PDUS and the MVD, and between the individual CEUS parameters and the MVD.

### Ethics approval and consent to participate

The study protocol adhered to the European Communities Council Directive (86/609/EEC)/principles of laboratory animal care (NIH publication No. 85-23, revised 1985). Approval was obtained from the local research Ethical Committee (Approval No. EC2015/143 and EC2016/66) of the Faculty of Veterinary Medicine of Ghent University, Belgium and from the Deontological Committee of the Federal Public Service of Health, Food Chain Safety and Environment, Belgium. Written informed consent was obtained from all patient owners before entry into the study. The use of CA4P in canine patients was approved (approval number 0002588) by the Belgian Federal Agency for Medicines and Health Products (FAMHP).

## Data Availability

The datasets used and/or analysed during the current study are available from the corresponding author on reasonable request.
